# Reliability of the Q Force; a mobile instrument for measuring isometric quadriceps muscle strength

**DOI:** 10.1186/s13102-016-0029-x

**Published:** 2016-02-19

**Authors:** K. W. Douma, G. R. H. Regterschot, W.P. Krijnen, G. E. C. Slager, C. P. van der Schans, W. Zijlstra

**Affiliations:** 1 Research and Innovation Group in Healthy Aging, Allied Health Care and Nursing, Hanze University of Applied Sciences Groningen, Groningen, The Netherlands; 2 Department of Rehabilitation Medicine, University of Groningen, University Medical Center Groningen, Groningen, The Netherlands; 3 University of Groningen, University Medical Center Groningen, Center for Human Movement Sciences Groningen, Groningen, The Netherlands; 4 School of Health Care Studies Hanze University of Applied Science Groningen, Groningen, The Netherlands; 5 Institute of Movement and Sport Gerontology, German Sport University, Cologne, Germany

**Keywords:** Quadriceps, Isometric muscle strength, Q force, Elderly

## Abstract

**Background:**

The ability to generate muscle strength is a pre-requisite for all human movement. Decreased quadriceps muscle strength is frequently observed in older adults and is associated with a decreased performance and activity limitations. To quantify the quadriceps muscle strength and to monitor changes over time, instruments and procedures with a sufficient reliability are needed. The Q Force is an innovative mobile muscle strength measurement instrument suitable to measure in various degrees of extension. Measurements between 110 and 130° extension present the highest values and the most significant increase after training.

The objective of this study is to determine the test-retest reliability of muscle strength measurements by the Q Force in older adults in 110° extension.

**Methods:**

Forty-one healthy older adults, 13 males and 28 females were included in the study. Mean (SD) age was 81.9 (4.89) years. Isometric muscle strength of the Quadriceps muscle was assessed with the Q Force at 110° of knee extension. Participants were measured at two sessions with a three to eight day interval between sessions. To determine relative reliability, the intraclass correlation coefficient (ICC) was calculated. To determine absolute reliability, Bland and Altman Limits of Agreement (LOA) were calculated and t-tests were performed.

**Results:**

Relative reliability of the Q Force is good to excellent as all ICC coefficients are higher than 0.75. Generally a large 95 % LOA, reflecting only moderate absolute reliability, is found as exemplified for the peak torque left leg of −18.6 N to 33.8 N and the right leg of −9.2 N to 26.4 N was between 15.7 and 23.6 Newton representing 25.2 % to 39.9 % of the size of the mean. Small systematic differences in mean were found between measurement session 1 and 2.

**Conclusion:**

The present study shows that the Q Force has excellent relative test-retest reliability, but limited absolute test-retest reliability. Since the Q Force is relatively cheap and mobile it is suitable for application in various clinical settings, however, its capability to detect changes in muscle force over time is limited but comparable to existing instruments.

## Background

Muscle strength is essential for all physical activities such as activities of daily living, work, sports and maintaining posture [1–4]. Reduced muscle strength is frequently apparent in older participants and creates a potential risk for a decline of activities. It may induce balance deficits, a risk of falling [3–10], and predisposition for disability, premature nursing home admission, and ultimately premature mortality [5, 11]. Also functional walking tests and rising tests, such as the timed up and go test, are used to predict the occurrence of future falls. Retrospective studies show that these tests are associated to a history of falls, the predictive capability however is limited [8, 11–13].

The quadriceps muscle is prominently important due to its major contribution to activities such as walking stairs, rising from a chair, and walking [3, 4, 7].

To quantify Quadriceps muscle strength and to monitor changes over a period of time, a muscle strength measurement with high reliability is required. Several viable quantitative muscle strength measurement methods are available. The Medical Research Counsel Scale (MRC) is the most commonly and clinically used method. The MRC scale ranges over 6 grades, 0 to 5. Unfortunately, this scale is inaccurate and inefficient in detecting changes over a period of time [14–18]. MRC grade 4 covers a wide range from 4 up to 99 % of the generated strength [19]. More precise measurements are possible with handheld dynamometry allowing muscle strength to be measured on a continuous scale. Handheld dynamometry has been demonstrated to have good reliability according to the intra class correlation coefficient of 0.8 or higher [19–22]. Measurements with a handheld dynamometer, however, are required to be performed in a reproducible joint position of 90° flexion [23–25]. Joint positions other than 90° flexion however, have exhibited greater maximal muscle strength values [26–28], as well as more extensive sensitivity when detecting changes over time [27]. For the knee the highest values were recorded by measurements at 110 and 130° extension and the most significant increase after training was determined in a knee angle position between 110 and 130° extension [28]. Another possible limitation of handheld dynamometry is the variance induced by different observers, i.e., certain patients restrain their efforts when working with presumed weaker observers. Consequently, fixation during measurement in healthy participants might be difficult [22, 23, 29]. The fixation possibilities of the observers are limited when using handheld dynamometry and this may negatively influence reliability and validity of muscle strength measurements [22, 23, 30]. An additional relevant aspect in any non-computerized type of muscle strength measurement is that it does not provide insight into coordination, slope, or duration of the contraction. Sustainable and repetitive contractions, however, are required in sports, and activities of daily living.

Another type of muscle strength measurement is isokinetic muscle strength measurement. Isokinetic equipment is capable of measuring from different body positions and angles, presenting an abundance of graphical, numerical, and derivative information which are considered to be the gold standard. The equipment, however, is expensive and immobile, and the procedure is time consuming.

The Q Force chair has been recently developed as a successor of the Quadriso Tester [31] to measure isometric muscle strength of the Quadriceps muscle in different joint angles. Advantages of the Q Force compared to other instrumentation is that it is transportable and can be employed easily in a clinical setting as for example a hand held dynamometer. It provides however as isokinetic instrumentation, relevant graphical, numerical and derivative information about the contraction besides measured peak values.

However, the test-retest reliability of the Q Force in older adults is unclear. Therefore the purpose of this study was to determine the test retest reliability of muscle strength measurements with the Q Force in older adults.

## Methods

The Medical Ethics Committee of the University Hospital Groningen approved this study.

All participants gave written informed consent before data collection began.

### Participants

In this study, inclusion criteria were:

*At least 70 years of age, being able to walk ≥10 m without support, and rise from a chair without resources or assistance;

*Absence of cardiovascular/respiratory or neurological disorders;

*No comorbidity or cognitive disorders that influence mobility, understanding, or execution of measurements. No current or recent participation in exercise programs or any other physical intervention;

*No orthopedic surgery or stroke within the last six months;

Participants were included after providing and signing informed consent. The Medical Ethics Committee of the University Medical Center Groningen, The Netherlands, approved the study protocol.

### Design

Muscle strength was assessed on two occasions within three to eight days. Four trials were performed on each occasion at approximately the same time of the day. If the participant was unable to perform four trials for any reason, fewer repetitions were performed and used for analysis

### Device

**Fig. 1:**
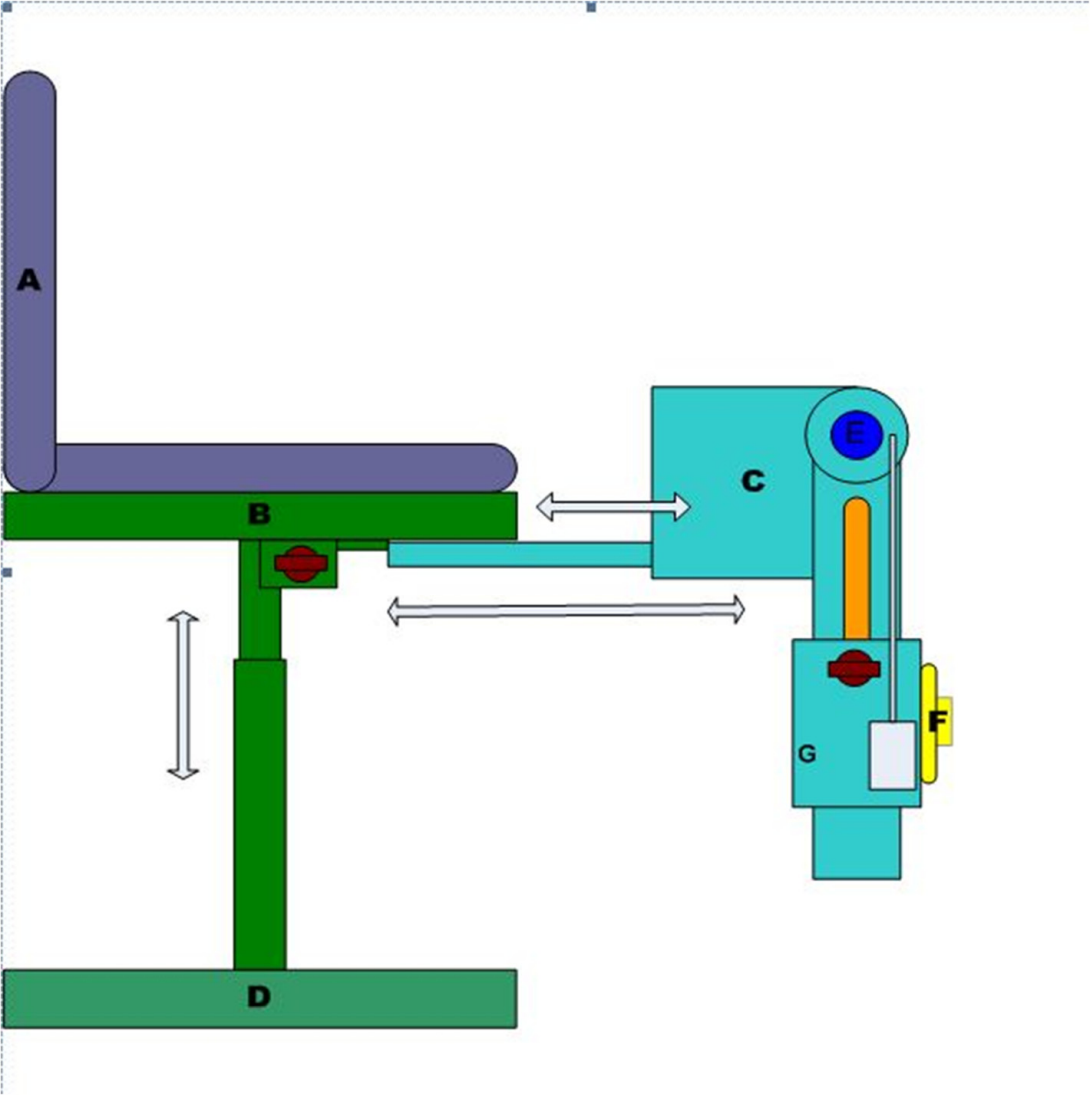
Schematic view, Q Force. **a** = Back support, **b** = Seat, **c** = Fixed brace, **d** = Base, **e** = Astrolabe/Goniometer, **f** = Force transducer, **g** = Distance transducer

**Fig. 2:**
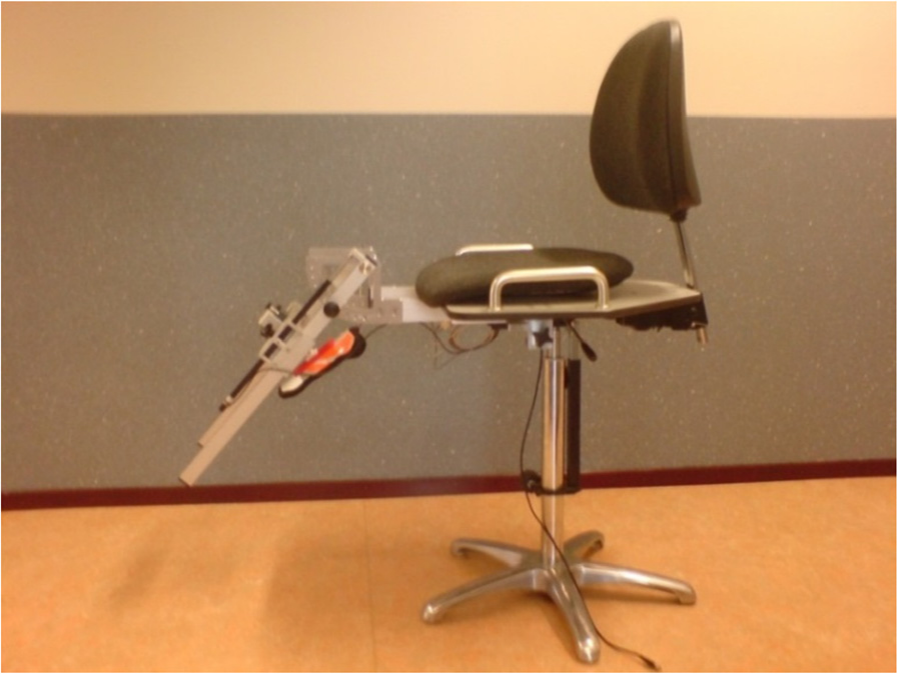
Q Force with fixed brace at the front

The Q Force (Fig. 1) has been constructed for measuring isometric Quadriceps muscle strength. It consists of a chair with an attached, adjustable fixed leg brace at the front (Fig. 2). Three sensors are located in the brace to determine the generated force (Newton); the angle between the horizontal chair surface and the brace and the distance between the force transducer and the rotation axle of the brace (millimeter). All three signals pass through an analog-digital converter, are read and saved on a laptop.

**Fig. 3:**
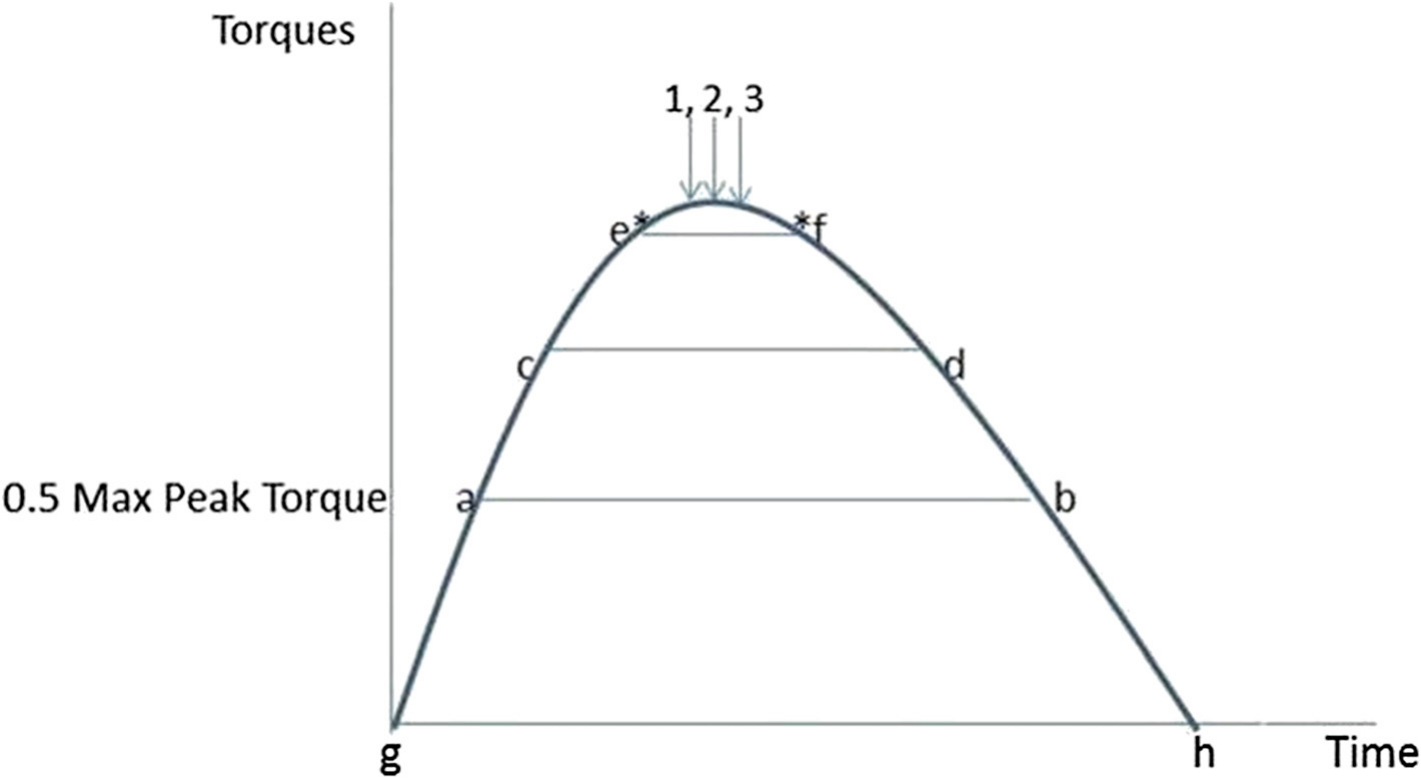
Graphic representation

The chair incorporates a solid frame, base, back support, and a seat. Bars are fitted at both sides of the seat for manual fixation. The height of the sitting surface is fully adjustable. At the left and right bottom of the seat, in an anterior posterior direction, rails are attached to which the brace is connected so that both the left and right leg can be tested. The fixed brace consists of a fixed horizontal and an adjustable distal component with a hinge in between; the brace can be slid horizontally via rails. The brace is adjustable to fit the subject’s upper leg dimensions. This affords placing the rotation axle of the brace in the same position as the rotation axle of the knee for that specific angle (Fig. 3). The measuring angle of the fixed brace is adjustable between 90° and 180°. The force transducer is covered with a pad to minimize pressure on the subject’s lower leg. The position of the pad is adjustable in vertical and horizontal directions in accordance with the subject’s dimensions.

### Computer and control

#### Hardware

The hardware consists of an analog-digital converter, a force, and a distance and angle transducer. The ADC is an NI-9219 4Ch Universal Analog input module. As an interface, the NI USB-9162 converter is utilized for establishing a USB connection between the ADC and the laptop. National Instruments Corporation Austin. The force transducer is an LLB400 Loadcell which is capable of measuring force up to 1100 Newton with a break load of 1650 Newton (Futek, Irvine). The distance transducer is a CLS1321 Linear potentiometer (Active Sensors Indianapolis). The angle is measured by a single strike potentiometer.

#### Software

The accompanying software is developed by the ICT Software Development Laboratory of the Faculty of Medical Sciences from the University of Groningen and the University Medical Center Groningen in the Netherlands.

### Outcome measures

We measured and calculated a peak value and three different average torque operationalization’s for each separated measurement; Peak Torque (PT), Filtered Peak Torque (FPT), Median Peak Torque (MPT) and Average Plateau Peak Torque (APT).

PT is the actually measured peak value and is calculated as Fmax * r + fixation torque. Fmax is the registered maximal force; r is the distance between the knee joint rotation point and the sensor on the lower leg; fixation torque is the torque required keeping the lower leg stabilized against gravity. Arrow 2 in Fig. 3 illustrates the PT value.

Filtered Peak Torque is the average peak torque of the sample that recorded the PT value [1] with the sample before (−1) and the sample after (+1) this sample and is calculated as $\mathrm{F}\mathrm{P}\mathrm{T}=\frac{\left( total\  torque\ \left(i-1\right)+ total\  torque\ (i)+ total torque\ \Big(i+1\right)\Big)}{3}$.

Arrow 1 and 3 in Fig. 3 indicate FPT.

**Fig. 4:**
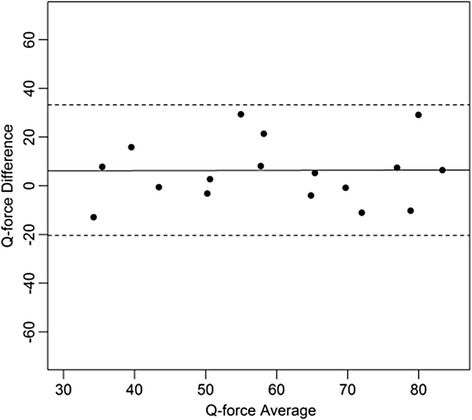
Limits of Agreement for mean Peak Torque measurements-left

MPT is defined as the median of the total torque above the level of 0.5 * PT. This is calculated as: median total torque = median (total torque (a:b)) whereby *a* is defined as the moment where total torque is greater than 0.5 * PT for the first time, and *b* is defined as the moment where total torque is smaller than 0.5 * PT for the first time. Line a-b in Fig. 4 represents 50 % of the PT level. Line c-d represents the MPT.

APT is the value above 50 % of the PT level. It is the average peak torque over the plateau phase. It is calculated as: average (total torque (c : d)) *c* is defined as the initial sample following sample *a* when the absolute difference with sample c −1 is less than 4 Nm. Sample *d* is defined as the sample following sample *a* where the absolute difference with sample d −1 is smaller than 4 Newtonmeter (Nm). Point *e* in Fig. 4 represents the first sample where the increase of the generated force is less than 4 Nm, and point *f* represents the last sample where the decline of the generated force *f* is less than 4 Nm. Line *e-f* represents the calculated value. This signifies that a plateau phase between samples c and d can be recognized in which the absolute differences between the sequential samples during this plateau are less than 4 Nm. This was considered a reliable contraction [30] representing the maximum generated torque. The level of elevation of this plateau corresponds with the generated torque. The mass and center of gravity of the lower leg were calculated according to Winter 1979 [32]. The sample ratio which recorded the generated forces was 2Hz.

Fig. 3 represents a graphic interpretation of the outcome measures. The vertical axis represents the generated torque while the horizontal axis represents the time. The letters g and h correspond with the start and the end of the contraction, a-b represents 05 * PT level, c-d represents the MPT and e-f represents the APT.

### Data acquisition

All algorithms for data acquisition were programmed and collected in Matlab Mathworks 7, Nathick, USA. The collected data were transferred to Microsoft Excel 2010 files for statistical analysis.

### Measurement procedure

The angle of the fixed leg brace was positioned at 110°, and the subject was subsequently positioned in the chair without back support and with the back of their knee positioned against the seat. The lateral condyle of the femur was aligned with the rotation point of the Q Force. The force transducer was positioned 3 cm above lateral malleolus. The tuberosity of the tibiae was aligned with the sensor to prevent adduction, abduction, or rotation in the knee or hip in the starting position. The computer program was initiated as the subject was instructed to elevate the leg to minimize pressure on the force transducer in order to calibrate the system. The distance between the transducer and the rotation point was determined to calculate the generated torque and the average knee angle. Additionally, the average distance from the rotation axle to the force transducer was measured to determine whether the current angle was actually 110°. Following the calibration, the actual measurement began whereby the left leg was tested first. Participants were allowed to fixate themselves to the sidebars using their hands. Four trials were performed for each leg. If the subject was not capable of performing four trials for any reason, fewer repetitions were performed and applied to the analysis. A 1 min break was administered between the successive trials. Following each trial, it was evaluated if the contraction had been maximal by asking, “Was this a maximal effort?” If the contraction had not been maximal, it was excluded.

### Instruction of the participant

The participants were instructed as follows: “Extend your knee as forceful as possible. I will measure the strength you will generate. You are allowed to fixate yourself to the side bars using your hands. Each leg is tested four times each with a one minute break in between. I will encourage you and tell you when to start and when to stop. The maximal contraction has to endure for three seconds. Build up your contraction gradually and let go slowly.

### Statistical analyses

All data were analyzed with the statistical programming language string R version 3.1.2 2014.

To determine relative reliability, the two-way absolute agreement variant of the intraclass correlation coefficient (ICC) was calculated. To determine absolute reliability the Bland and Altman limits of agreement (LOA) were calculated according to 1.96 * SD of the mean difference [33]. The LOA were calculated for each pair of measurements and were interpreted with regard to the clinical relevance to their size.

Bland and Altman plots were constructed with the difference and the mean of the of the two measurements for each subject. Means, standard deviation, mean difference, and standard deviations were calculated for descriptive purposes. The paired t-test was used to test for systematic differences in mean between session 1 and session2. ICCs were interpreted as: ICC < 0.25 low; 0.25 < ICC < 0.50 moderate; 0.50 < ICC < 0.75 moderate to good; and ICC > 0.75 is excellent reliability [34–36]. A level of 0.05 was considered significant.

## Results

### Participants

Forty-one healthy older adults were included in the study comprised of 13 males and 28 females. Mean (SD) of age was 81.9 (4.89) years; of body weight was 78.5 (13.0) kg; and of body height 165.3 (5.8) cm.

### Outcome measures

Table 1 presents the reliability results of session1 and session 2 for the left and right leg.

**Table 1:** Shows the intraclass correlations, the t-test outcomes and the LOA’s for the left and right leg

		Session 1	Session 2	diff		*P* value			
		Mean (SD)	Mean (SD)	Mean (SD)	t-test	ICC	L. Loa	U. Loa	Loa % Mean
PT	left	63.1 (27.2)	70.7 (29.2)	7.6 (13.4)	0.0078	0.89	−18.6	33.8	39.1
PT	right	66.1 (30.4)	74.8 (34.2)	8.6 (9.1)	0.0001	0.96	−9.2	26.4	25.1
FPT	left	62.5 (27.0)	70.2 (29.1)	7.7 (13.5)	0.0077	0.88	−18.8	34.2	39.9
FTP	right	65.6 (30.3)	74.0 (34.1)	8.4 (9.1)	0.0001	0.96	−9.4	26.2	25.5
MPT	left	58.5 (26.0)	66.4 (28.5)	7.9 (12.5)	0.0037	0.89	−16.6	32.4	39.2
MPT	right	62.0 (29.1)	69.9 (32.9)	7.9 (9.0)	0.0001	0.96	−9.7	25.5	26.7
APT	left	58.3 (26.2)	65.9 (28.6)	7.6 (12.9)	0.0058	0.80	−17.6	32.8	38.7
APT	right	61.8 (29.6)	70.2 (33.5)	8.4 (9.1)	0.0001	0.96	−9.4	26.2	26.9

*PTL* Peak Torque, *FTP* Filtered Peak Torque, *MPT* Median Peak Torque, *APT* Average Plateau Peak Torque, expressed in Newton, * Significant level; ≤ 0.01, ICC; Intraclass Correlation Coefficient, L.LOA; Lower Limits of Agreement, U. LOA upper Limits of Agreement. LOA as percentage of mean of session1 and session 2

All differences between Session1 and Session2 are significant according to the Paired T-Test. It can be observed from Table 1 that the ICC coefficients are higher for the right than for the left leg and all ICC coefficients are greater than 0.75. The LOA’s are smaller for the right leg compared to the left and are in general relatively large, 17.6 to 26.5 Newton, and represent values between 25.2 % to 39.9 % of the mean measured values of session 1 and session2. The LOAs are smaller for the right than for the left leg.

Figures 4, 5, 6, 7, 8, 9, 10 and 11 present Bland and Altman plots of the limits of agreement between session1 and session2 measurements of the mean Peak Torque; the mean Median Peak Torque; the mean Plateau Peak Torque; and the mean Filtered Peak Torque of the left and right leg, respectively.

**Fig. 5:**
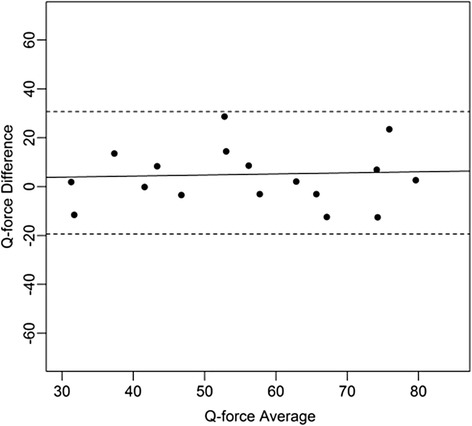
Limits of Agreement for mean Median Peak Torque measurements-left

**Fig. 6:**
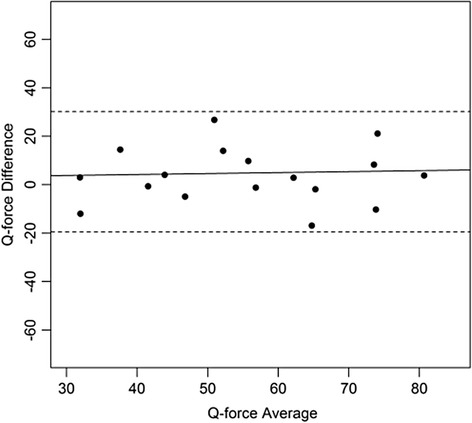
Limits of Agreement for meanPlateau Peak Torque measurements-left

**Fig. 7:**
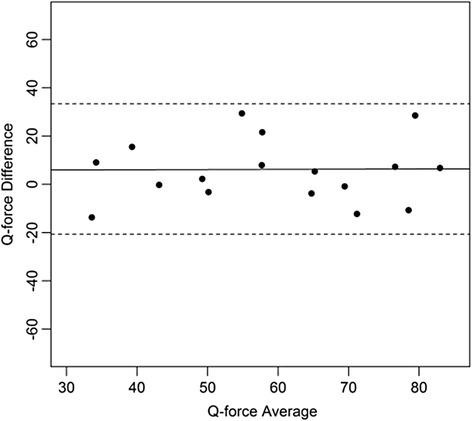
Limits of Agreement for mean Filtered Peak Torque measurements-left

**Fig. 8:**
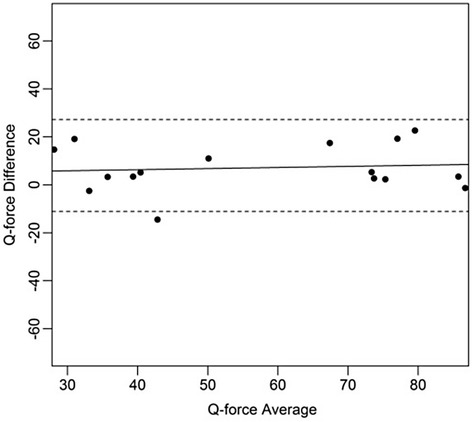
Limits of Agreement for mean Peak Torque measurements-right

**Fig. 9:**
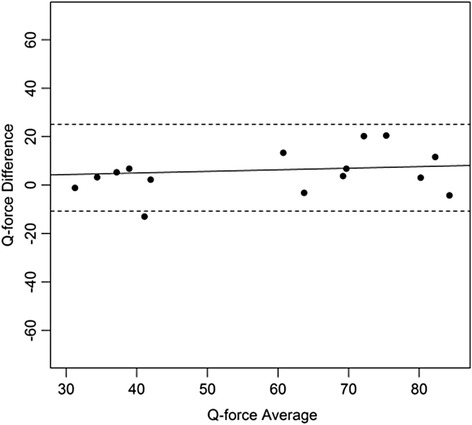
Limits of Agreement for mean Median Peak Torque measurements-right

**Fig. 10:**
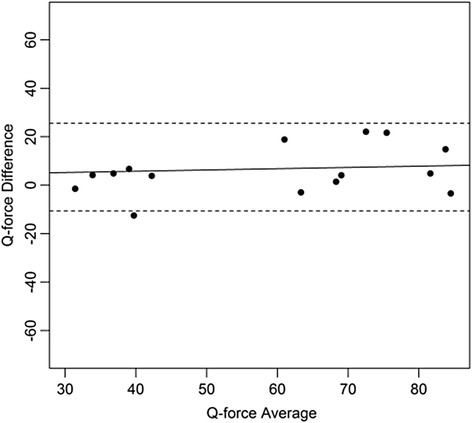
Limits of Agreement for mean Plateau Peak Torque measurements-right

**Fig. 11:**
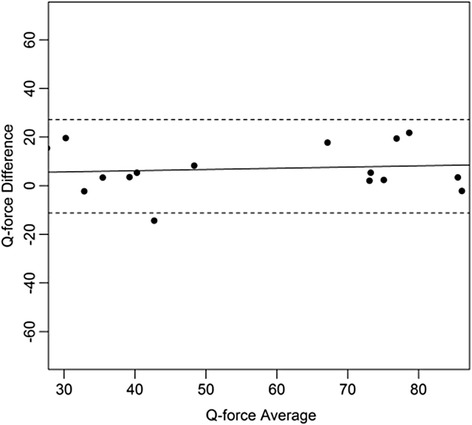
Limits of agreement for mean Filtered Peak Torque measurements-right

The uninterrupted horizontal line is located above zero in all cases due to the systematic difference between Session1 and Session2. The variation of all of the measurements is not increasing with increasing Q Force torque, and there are only minimal outlying points observed. The right leg measurements result in smaller LOA than those for the left.

## Discussion

The outcomes of this study indicate that muscle strength measurements with the Q Force at 110° flexion of the knee are reliable according to the ICC coefficients. The ICC coefficients exceed 0.75 which indicate excellent relative test-retest reliability [34–36]. However, the obtained LOA’s are substantial indicating moderate absolute reliability. The mean values at the second measurement are significantly higher than those at the first measurement. The encountered ICC and LOA presented are consistently better for the right leg compared to the left.

The obtained ICC coefficients indicate that the Q Force is capable of reliably measuring muscle strength on group level [34–36]. The encountered ICC coefficients are between 0.80 and 0.95 and therefore consistent with coefficients determined in other muscle strength measurement reliability studies [20–23, 29, 37, 38, 40, 41]. The ICC coefficients computed over the outcome measures, Peak 8Torque (PT), Filtered Peak Torque (FPT), Median peak Torque (MPT) and Average Plateau Peak Torque (APT) are very comparable, indicating that the relative reliability can be generalized over different types of muscle strength measures. We measured muscle strength at 110° knee extension. Studies based on different degrees of extension ascertained ICC coefficients between 0.87 and 0. 99 and are in accordance with our findings [20–23, 29, 37–41] This suggests that the reliability of Q Force measurements can be generalized to measurements other than 110° extension.

ICC coefficients provide information regarding relative test-retest reliability of the instrument. The ICC coefficients do however not provide information on the magnitude of the intra individual variation between two observations [42]. These intra individual variations are represented by the absolute reliability as expressed by the limitis of agreement (LOA) . The overall variation summarized in the LOA can be influenced by several sources of variation such as the time of the day, type of measurement, subject, observer, and protocol. The LOAs we found are substantial and vary between 15.7 and 23.6 Nm which corresponds to 22.5 % and 36 % of the mean. This magnitude of the LOA’s is consequential for clinical or research practice since a true change after training, for example, can only be detected if it is at least 22.5 % in magnitude. Other studies with different type of instrumentation and different populations also describe a limited absolute reliability [20, 32, 38, 43, 44]. This indicates that though the clinical usefulness of the Q Force for measuring muscle strength, the ability to detect changes over time is limited, although quite comparable with other instruments. In addition, the measurements outcomes for the right leg presents smaller LOA than the left. The different LOA size between the left and right leg can possibly be explained by differences in individual variation due to the fact that the majority of a population is right-side dominant [44]. Right dominance might result in a increased neural drive to the dominant right leg resulting in more consistent values. However differences in ICC and LOA values between left and right are not always found in comparable studies.

By the results of the paired T-test we found systematic differences between the first and second session in the sense that means on the second are approximately ten percent higher compared to the first session. Other studies using isokinetic devices or pre trials prior to the actual measurement also present systematic differences or a tendency to higher values at the second measurement session [45–50]. Several studies suggest that fear, a distinct learning effect or increased muscle recruitment may be responsible for systematic differences [20, 28, 46–50].

In our study we tested a population of healthy older adults. It does not provide any information about measurements reliably for example a chronically ill population or healthy working adults. We did not register if people were left or right dominant. This limits de insight in the origin of the observed difference in ICC, LOA between left and right. We did not perform pre trials at session 1 which might contribute to the observed differences between the measured values during session 1 and 2. Though strength measurements reflect the quadriceps muscle force however no reliable procedures are available translating muscle strength into function. Therefore interpretation of strength measurement should be performed with caution.

Though muscle strength is often decreased in older subjects and associated with balance deficits and risk of falling, it is probably insufficient to use only muscle strength measurements in the predicting of fall incidents. A combination of measurements of muscle strength and functional rising and walking tests may increase the predictive capacity considerably.

## Conclusion

Q Force measurements in 110° extension have excellent absolute reliability (ICC), but only moderate absolute reliability (LOA). The size of the latter indicates a limited capability to detect changes in muscle force over time. Since the Q Force is relatively cheap and mobile it seems suitable for application in various clinical settings. Future studies should investigate the degree in which its discriminative ability can be improved especially in older adults.

## Abbreviations

APT: average plateau peak torque
FPT: filtered peak torque
ICC: intraclass correlation coefficient
LOA: limits of agreement
MPT: median peak torque
MRC: medical research council scale
PT: peak torque
